# Assessment of COVID-19 RT-PCR Positive Symptomatic Patients With Clinical, Hematological, and Radiological Parameters Among Three Groups: A Comparative Study

**DOI:** 10.7759/cureus.39681

**Published:** 2023-05-30

**Authors:** Natesh G, Anbumaran Parivakkam Mani, Gangadharan Vadivelu, Preethi Selvaraj, Sankalp Yadav

**Affiliations:** 1 Respiratory Medicine, Saveetha Medical Collage and Hospital, Saveetha Institute of Medical and Technical Sciences, Kancheepuram, IND; 2 Community Medicine, Sri Lalithambigai Medical College and Hospital, Chennai, IND; 3 Medicine, Shri Madan Lal Khurana Chest Clinic, Moti Nagar, New Delhi, IND

**Keywords:** erythrocyte sedimentation rate (esr), c –reactive protein (crp), rtpcr, covid-19, covid-19 india

## Abstract

Background: Nearly 70.1 million individuals have been infected by the pandemic viral disease known as coronavirus disease 2019 (COVID-19), which was first discovered in China and is caused by a novel coronavirus known as severe acute respiratory syndrome coronavirus 2 (SARS‑CoV‑2). This disease is responsible for the deaths of 6 million people. India ranks third in the total number of cases. The purpose of this study was to classify COVID-19 patients according to several criteria and to determine which clinical, hematological, and radiological indicators were most important in their care.

Materials and methods: An analytical cross-sectional study was conducted on a total of 70 symptomatic patients who tested positive for COVID-19 reverse transcription polymerase chain reaction (RT-PCR) and were hospitalized at the Saveetha Medical College and Hospital in Chennai, Tamil Nadu, India, for the duration of the study. Comorbidities and oxygen reliance were taken into consideration while classifying patients into one of three categories. Initial symptoms, as well as hematological (interleukin-6 (IL-6), erythrocyte sedimentation rate (ESR), c-reactive protein (CRP), D-dimer, serum ferritin, and total cell counts) and radiographic (X-ray and computed tomography (CT) of the thorax) characteristics, were taken and analyzed among the different groups.

Results: According to our research, the symptom of fever was the most common, accounting for 84.3% of all cases. This was followed by breathlessness (55.7%), myalgia (31.4%), dry cough (27.1%), sore throat (24.3%), cough with expectoration (20%), loose stools (12.9%), loss of taste (12.9%), and smell (11.4%). Although there was a large amount of variation in D-dimer, with Category C having the highest values, there was only a minor amount of variation in ESR and CRP. The X-ray and CT scans of the chest showed substantial differences between the groups, with CT findings such as COVID-19 Reporting and Data System (CO-RADS) and CT severity score, consolidation, crazy paving pattern, and vascular dilatation showing a wide range of differences between the groups.

Conclusions: To facilitate easier treatment and place more attention on radiological characteristics using D-dimer, treating physicians are required to categorize COVID-19 patients into several groups. Patients who need oxygen support were included in this category.

## Introduction

Coronavirus, often known as severe acute respiratory syndrome coronavirus 2 (SARS‑CoV‑2), is a ribonucleic acid (RNA) virus that poses a significant risk to people's health all over the world. Over the course of the last 10 years, SARS-CoV-2 has become the third known coronavirus to be responsible for fatal respiratory illnesses in people. It is speculated to be transmitted from bats to humans via a reservoir/amplifier (an unknown animal). Several susceptibility studies have shown that domestic cats, ferrets, hamsters, and minks are also susceptible to infection and could serve as intermediate animal host species and new sources for spillover events into the human population. Similarly, transmission from infected humans to animals has also been reported [1]. The clinical spectrum of SARS-CoV-2 includes both cases in which patients do not exhibit any symptoms and patients who do exhibit mild, moderate, or severe symptoms, with or without pneumonia. Nearly 70.1 million people have been impacted by it, and it has been responsible for the deaths of 6 million people. India is ranked third in terms of the total number of cases [2]. This study aims to compare the hematological and radiological patterns of coronavirus disease 2019 (COVID-19) reverse transcription polymerase chain reaction (RT-PCR)-positive patients among three groups to find the significant inflammatory markers in treating COVID-19 patients and to observe the important radiological signs and correlate them with COVID-19 disease severity. Additionally, this research seeks to find significant inflammatory markers in treating COVID-19 patients.

## Materials and methods

After obtaining institutional ethical clearance [SCAHS/IRB/2021/May/080 dated 06.05.2021], an observational and comparative study was conducted among 70 COVID-19 RTPCR-positive, symptomatic patients who were admitted to the Saveetha Medical College and Hospital in Chennai, Tamil Nadu, between September 2021 and December 2021. Patients who were older than 21 years old and patients who tested positive for COVID-19 RT-PCR were included in the study. Patients with COVID-19 who had previously been treated for the disease or who had a radiological diagnosis of the disease were not included in the study (Table 1).

**Table 1:** Inclusion and exclusion criteria of the study subjects COVID-19: Coronavirus disease 2019. RT-PCR: Reverse transcription polymerase chain reaction.

Inclusion criteria:
Patients aged 21 years or older
Patients with a positive result for the COVID-19 RT-PCR test
Patients who were symptomatic and admitted to Saveetha Medical College and Hospital in Chennai, Tamil Nadu, between September 2021 and December 2021
Patients who provided written informed consent to participate in the study
Exclusion criteria:
Patients who had previously received treatment for COVID-19
Patients who had a radiological diagnosis of COVID-19 before admission
Patients who had other comorbidities that could potentially confound the interpretation of hematological and radiological parameters
Patients who were pregnant
Patients who were unable to provide informed consent
Patients who were unable to undergo hematological or radiological testing due to medical contraindications or other reasons

The clinical records with age, sex, comorbidities, and symptoms of patients with positive hematological parameters (interleukin-6 (IL-6), D-dimer, c-reactive protein (CRP), erythrocyte sedimentation rate (ESR), total white blood cell count (TC), differential count (DC), random blood sugar (RBS), HbA1C, serum ferritin), and radiological parameters based on chest X-ray and CT thorax were monitored and recorded. Patients were categorized based on their vitals and comorbid status into groups A, B, and C (Table 2). Group A consisted of patients with no comorbidities; Group B consisted of patients with one comorbidity; and Group C consisted of patients with two or more comorbidities or without any comorbidity. 

**Table 2:** Categorization of patients based on their vitals and comorbid status SpO_2_: Saturation of peripheral oxygen

Parameters	Group A	Group B	Group C
Pulse (per min)	60 – 100	100 – 120	> 120
Systolic blood pressure (mm Hg)	> 120	100 – 120	< 100
Diastolic blood pressure (mm Hg)	> 80	60 – 80	< 60
Respiratory rate (per minute)	< 18	18 – 24	> 24
SpO_2_ (percent)	> 94	> 94	< 94
Co-morbidities (diabetes, tuberculosis, chronic obstructive pulmonary disease, asthma, chronic kidney disease, cancer, hypertension.)	Absent	Present	Present (more than 1 comorbidity) or Absent

Statistical analysis

Using IBM Corp. Released 2019. IBM SPSS Statistics for Windows, Version 26.0. Armonk, NY: IBM Corp, the acquired data were evaluated. The data were summarized using descriptive statistics and frequency analysis, with continuous variables using the mean and standard deviation and categorical variables using percentages. Tukey's posthoc test was used in conjunction with a one-way analysis of variance (ANOVA) to identify any significant differences. The chi-square test was also employed to see if the category data had any significance. A probability value of 0.05 is regarded as a significant threshold in each of these tests.

## Results

**Figure 1:**
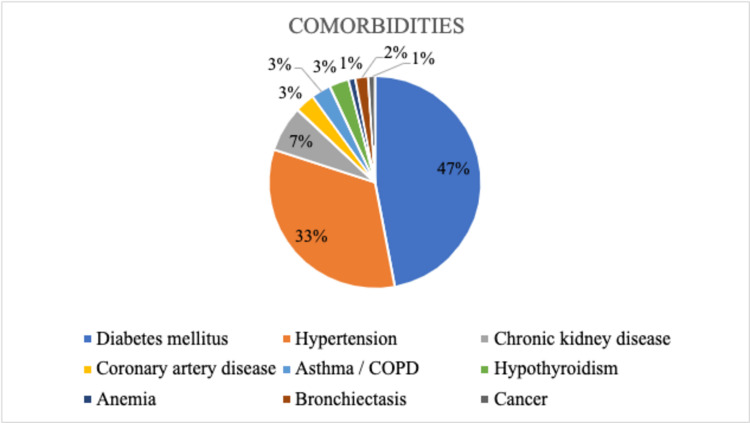
Distribution of comorbidities among the study population

In our study population of 70 patients, Group A consisted of 12 patients, Group B of 24 patients, and Group C of 34 individuals following categorization. Group A's median age was 31 years, Group B's was 55 years, and Group C's was 59 years. Males (N = 41) made up more of the study's population than females (N = 29). In our study, fever (84.3%), dyspnea (55.7%), myalgia (31.4%), a dry cough (27.1%), a sore throat (24.3%), a cough with expectoration (20%), loose stools (12.9%), and a loss of taste and smell were the most prevalent symptoms (11.4 %). In contrast to the other two groups, group C patients' breathlessness (p-value: 0.002) was significant. Most patients were diabetic (47%), followed by hypertensive (33%), and had a few other comorbidities (Figure 1).

**Figure 2:**
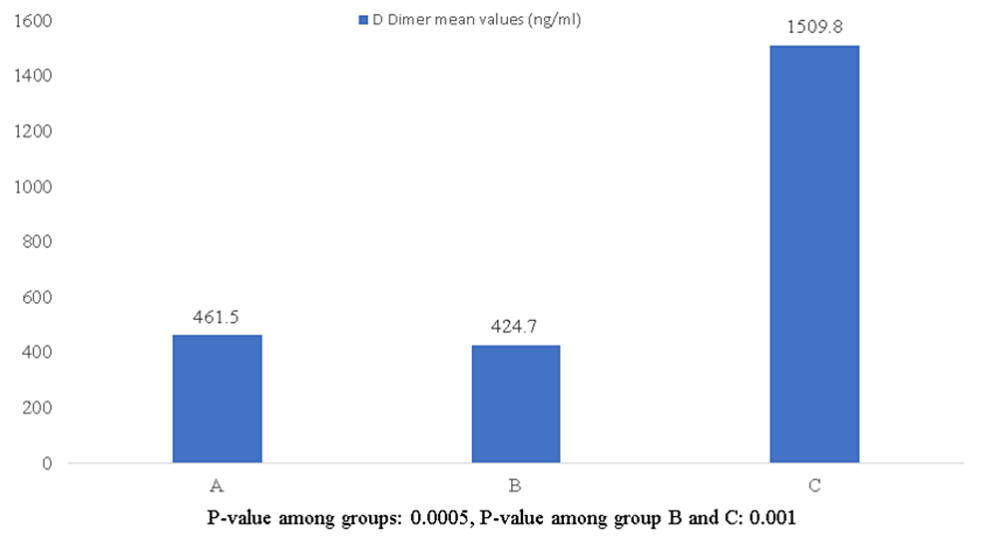
Mean D-dimer values among three groups of patients

Patients in Group C had significant desaturation, and the mean saturation was 84%. Comparing blood parameters among the three groups, D-dimer was elevated significantly in Group C patients (Figure 2) and was statistically significant (p-value 0.005).

**Figure 3:**
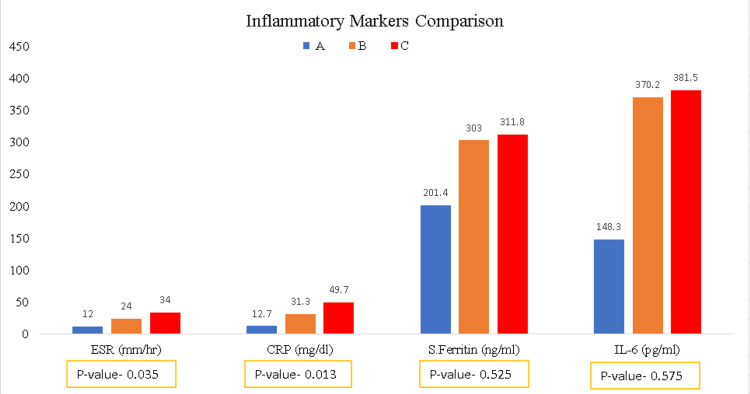
Comparison of inflammatory mediators among groups (ESR and CRP have statistical significance, while serum ferritin and IL-6 do not) ESR: Erythrocyte sedimentation rate. CRP: C-reactive protein. S. Ferritin: Serum ferritin. IL-6: Interleukin-6.

Inflammatory markers like CRP and ESR were elevated in all groups of patients and were statistically significant (p-values of 0.035 and 0.013, respectively) (Figure 3).

**Figure 4:**
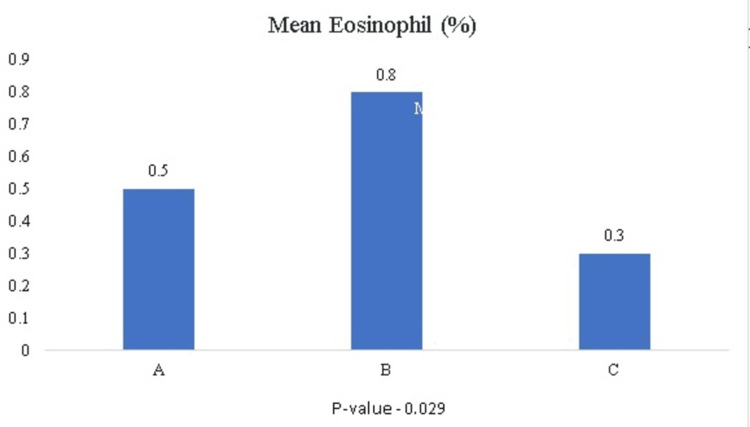
Mean eosinophil percent among three groups

Other inflammatory mediators like IL-6 and serum ferritin, though elevated, showed no statistical significance (p-values of 0.52 and 0.57, respectively). Total counts were elevated among all three groups, with mean total counts higher than 10,000 in all three groups, while eosinophils were significantly decreased in patients requiring oxygen support (Figure 4) and were statistically significant (p-value 0.029). 

**Figure 5:**
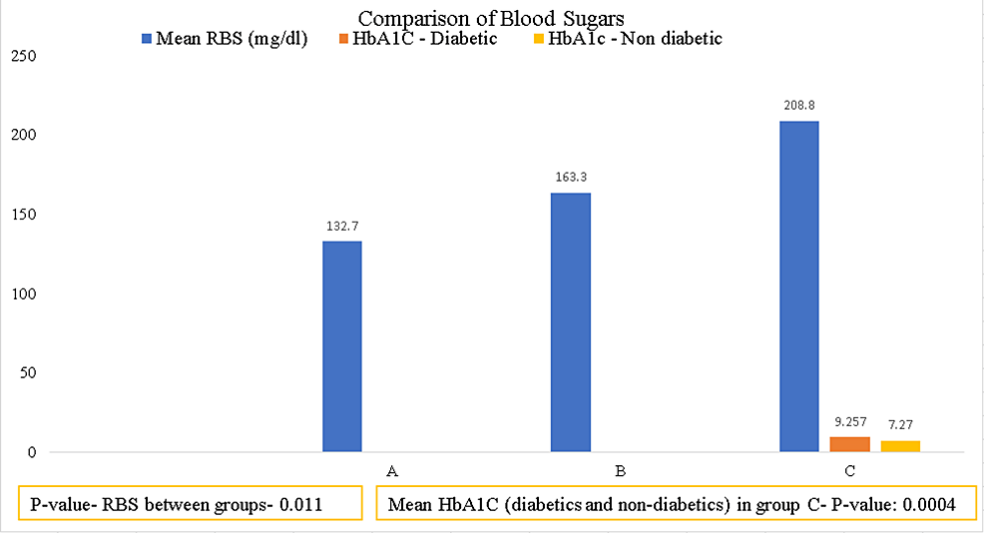
Mean RBS values among groups and mean HbA1C values among diabetics and non-diabetics in group C RBS: Random blood sugar

Patients in group C had an increased mean RBS (Figure 5), and the HbA1C comparison between diabetics and non-diabetics in group C was high and statistically significant (p-values 0.011 and 0.0004).

It was significantly easier to diagnose the severity of COVID-19 using radiology imaging. When compared to a chest X-ray, computerized tomography of the thorax was substantially more sensitive. Patients with severe COVID-19 had higher rates of some CT thorax abnormalities, including consolidation, vascular dilatation, and crazy paving patterns (Table 3).

**Table 3:** Chest radiography and CT thorax findings among three groups CT thorax: Computed tomography of the thorax

Chest X-ray				
Radiological findings	Group A	Group B	Group C	p-value
No evidence	8	8	0	0.015
Opacities	4	16	34	
CT-Thorax				
Severity Score: Mild (<8)	7	11	0	0.0001
Severity Score: Moderate (9-15)	5	8	13	
Severity Score: Severe (>15)	0	5	21	
Consolidation	1	4	23	0.0005
Crazy paving pattern	6	11	28	0.009
Vascular dilatation	6	13	31	0.002
Sub-pleural bands	6	12	20	0.760
Traction bronchiectasis	0	3	8	0.136

**Figure 6:**
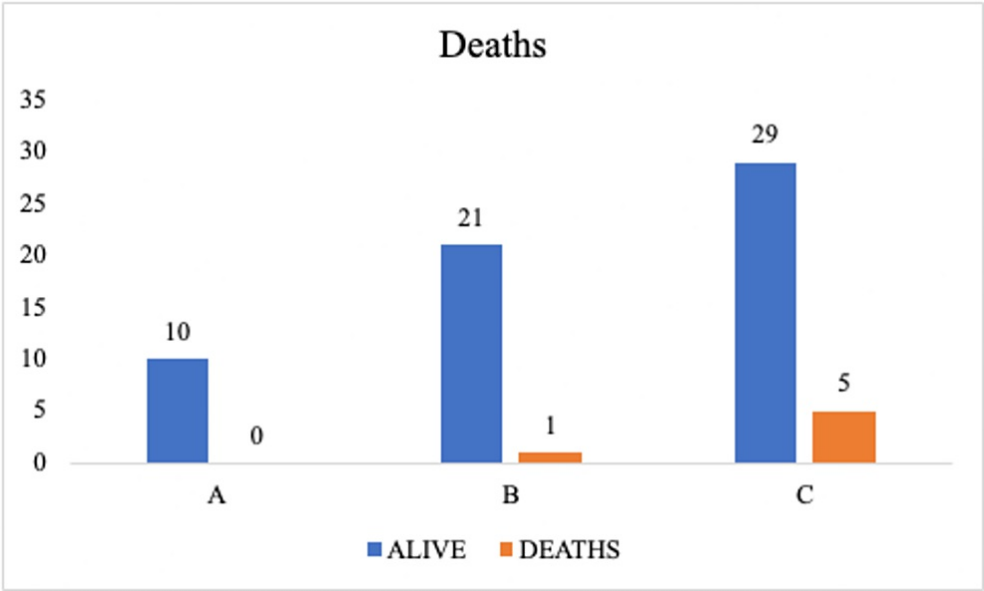
Number of deaths among the groups

Six individuals out of 70 died from COVID-19; the majority of them had a severe form of the disease (Figure 6).

## Discussion

This study showed that people with comorbidities had a higher chance of requiring oxygen support. In comparison among the three groups, breathlessness was significantly greater in group C patients.

D-dimer was elevated in patients requiring oxygen support, indicating severe disease. Various studies also indicate D-dimer was elevated with increased severity of the disease and was a major risk factor for poor prognosis and an elevated hospital mortality rate. Newer studies also highlight the use of anti-coagulants, which decreased the post-COVID-19 mortality rate [3]. Although the precise cause of elevated D-dimer is unknown, Guler Neil proposed that it may be due to uncontrolled apoptosis of alveolar endothelial cells brought on by a virus that can impair oxygenation through the formation of thrombi in the alveolar vascular bed and vascular fluid leaks into lung tissue [4]. Additionally, there is growing clinical proof that cytokine storms initiate a coagulation cascade that results in thrombotic complications in severe COVID-19 cases [5].

Inflammatory markers like CRP and ESR were elevated among all groups and had significant variation among groups. IL-6, a pro-inflammatory cytokine and a marker for acute inflammation, though elevated, failed to identify patients with severe COVID-19. Although several studies indicate elevated IL-6 to be a poor prognostic factor, this study shows IL-6 to be elevated irrespective of patient status [6]. This might be due to the initial high viral load causing damage to the lower respiratory tract [7]. Similarly, in this study, serum ferritin, though elevated, showed no significant variation among groups. A meta-analysis study showed marked elevations in inflammatory markers such as ESR, CRP, serum ferritin, and IL-2, -6, and -10 in patients with severe COVID-19, which were also noted in this study [8]. Due to the paucity of funding, we only took inflammatory marker panels available in our institutes, like IL-6, ESR, CRP, and serum ferritin.

Total leukocyte counts were elevated in all three groups. Although several studies indicate elevated total counts in COVID-19 patients, this study showed no significant variation among groups [9]. In this study, we found a decreased eosinophil count among patients requiring oxygen support. The precise mechanism of eosinopenia is currently unknown. There are various mechanisms, like decreased production from the bone marrow, increased margination within blood vessels, increased migration to somatic tissues, and decreased survival in peripheral blood circulation [10]. However, several studies indicate that cytokine storm, which causes a depletion of eosinophils, is a probable potential mechanism of eosinopenia [11,12]. Two scoring systems were also introduced, COVID-19-REAL and PARIS risk assessment tools, in which elevated eosinophils were among the hematological parameters to identify patients who would have COVID-19 as a diagnosis [13,14].

RBS and hemoglobin A1C (HbA1C) increased among diabetics in Group C. In a study on 180 COVID-19 patients, it was shown that high HbA1C levels were associated with high mortality among diabetics [15]. Various studies also suggest diabetics have a poor prognosis and higher mortality [16,17]. The disease severity and mortality among diabetics could be due to decreased immunity. Previous coronavirus infections like severe acute respiratory syndrome (SARS) and Middle East respiratory syndrome (MERS) also showed a similar picture of increased mortality and severity in diabetics [16]. The use of corticosteroids is both a boon and a bane, useful in controlling cytokine storms but, on the other hand, raising the blood sugar levels of diabetics [18,19]. Diabetics taking steroids are more vulnerable to opportunistic infections due to weakened immune systems. 

Chest radiography in this study was useful in detecting cases from low socioeconomic backgrounds and had high sensitivity in the diagnosis of COVID-19 patients requiring oxygen. Various studies have shown chest X-rays have no clinically meaningful association with symptoms or vital signs [20]. The chest X-ray can only be used in triage at initial presentation and is useful in categorizing patients with extensive disease. Chest X-ray changes were predominant in older people and patients requiring oxygen. Various comparison studies between the diagnostic accuracy of chest X-ray versus CT thorax have shown the CT thorax to have high sensitivity in assessing the extent of severity and lung involvement [21]. Serial chest X-rays can be used to monitor the prognosis of patients, thus limiting their greater exposure to ionizing radiation during CT thorax and in patients who are on mechanical ventilation.

Various studies have shown CT-thorax to be the most sensitive method for diagnosing COVID-19 pneumonia. Most studies like this one show a lower lobe predominance and a higher CT score correlated with the patient’s elevated D-dimer, inflammatory markers, and disease severity [22]. CT scoring systems like COVID-19 reporting and data system (CO-RADS) and CT severity scores were useful in inter-observer agreements for ease of understanding and accessing the severity and prognosis of the disease [23]. There are various scoring systems for CT thorax like CO-RADS, the Radiological Society of North America (RSNA) expert consensus statement, and the British Society of Thoracic Imaging (BSTI) guidance statement and a study comparing different scoring systems showed reasonable performance [24-27]. CT findings like consolidation, vascular dilatation, and crazy paving patterns were more significant in people requiring oxygen support. The above findings were consistent with various studies [28]. CT thorax findings in correlation with hematological parameters help in assessing the patient's prognosis and need for escalation of treatment. 

The study has limitations, as it was done at a single center, and therefore it's imperative that data from different parts of the world be collected for a deeper insight into the aims addressed in the present study.

## Conclusions

COVID-19 patients must be categorized by treating physicians for convenience of care. When treating individuals who need oxygen support, the inflammatory markers CRP and ESR are important. HbA1C may help track inflammation, hypercoagulability, and prognosis in COVID-19 patients who have diabetes. Patients who need oxygen assistance should be given special attention using radiological parameters and D-dimer as diagnostic and therapeutic tools.
